# In vivo imaging of the immune response upon systemic RNA cancer vaccination by FDG-PET

**DOI:** 10.1186/s13550-018-0435-z

**Published:** 2018-08-15

**Authors:** Stefanie Pektor, Lina Hilscher, Kerstin C. Walzer, Isabelle Miederer, Nicole Bausbacher, Carmen Loquai, Mathias Schreckenberger, Ugur Sahin, Mustafa Diken, Matthias Miederer

**Affiliations:** 1 Department of Nuclear Medicine, University Medical Center Mainz of Johannes Gutenberg University Mainz, Mainz, Germany; 2 TRON - Translational Oncology at the University Medical Center of Johannes Gutenberg University Mainz gGmbH, Mainz, Germany; 3 Biopharmaceutical New Technologies (BioNTech) Corporation, Mainz, Germany; 4 Department of Dermatology, University Medical Center Mainz of Johannes Gutenberg University Mainz, Mainz, Germany

**Keywords:** FDG PET/MRI, Small animal PET, Cancer vaccination, RNA-lipoplex

## Abstract

**Background:**

[^18^F]Fluoro-2-deoxy-2-d-glucose positron emission tomography (FDG-PET) is commonly used in the clinic for diagnosis of cancer and for follow-up of therapy outcome. Additional to the well-established value in tumor imaging, it bears potential to depict immune processes in modern immunotherapies. T cells enhance their glucose consumption upon activation and are crucial effectors for the success of such novel therapies. In this study, we analyzed the T cell immunity in spleen after antigen-specific stimulation of T cells via highly innovative RNA-based vaccines using FDG-PET/MRI. For this purpose, we employed systemic administration of RNA-lipoplexes encoding the endogenous antigen of Moloney murine leukemia virus (gp70) which have been previously shown to induce potent innate as well as adaptive immune mechanisms for cancer immunotherapy. Feasibility of clinical imaging of increased splenic FDG uptake was demonstrated in a melanoma patient participating in a clinical phase 1 trial of a tetravalent RNA-lipoplex cancer vaccine.

**Results:**

We observed exclusive increase of glucose uptake in spleen compared to other organs thanks to liposome-mediated RNA targeting to this immune-relevant organ. In vivo and ex vivo FDG uptake analysis in the spleen of vaccinated mice correlated well with antigen-specific T cell activation. Moreover, the use of an irrelevant (antigen non-specific) RNA also resulted in enhanced FDG uptake early after vaccination through the activation of several other splenic cell populations. The glucose uptake was also dependent on the dose of RNA administered in line with the activation and frequencies of proliferating antigen-specific T cells as well as the general activation pattern of splenic cell populations.

**Conclusions:**

Our preclinical results show rapid and transient vaccination-induced increase of FDG uptake within the spleen reflecting immune activation preceding T cell proliferation. FDG-PET/CT in patients is also capable to image this immune activation resulting in a new potential application of FDG-PET/CT to image immune processes in new immunological therapies.

**Electronic supplementary material:**

The online version of this article (10.1186/s13550-018-0435-z) contains supplementary material, which is available to authorized users.

## Background

Taking advantage of the natural potential of the immune system to fight against cancer in different clinical stages led to major milestones in immune-mediated cancer therapy. Among the many pathways that now can be addressed by systemic drug delivery, the delivery of tumor antigen encoding RNA is particularly promising. Through this platform technology, transient genetic programming of target antigen-presenting cells is possible without more complicated introduction of genetic information via plasmid DNA, and a broad repertoire of T cells is recruited in patients treated with in vitro transcribed RNA for neo-epitope vaccination [1]. Moreover, novel methods overcoming the typical rapid RNA degeneration via RNases encapsulation of RNA into nanoparticular structures led to innovative therapeutic approaches opening a new field in cancer therapy [2]. Systemic RNA application accomplished by RNA complexed liposomes (RNA-lipoplexes) induces two—in principle—distinct effects: One is (rapid) activation of immune cells by stimulation of toll-like receptors (TLR, in particular TLR7), and the other is induction of proliferation of specific CD8^+^ T cells reactive against the translated protein. These T cells—programmed to attack tumor cells—bear the high promise in new cancer vaccination therapies [2]. RNA-based immune interventions by means of nanoparticular-based targeting of immunologically relevant organs such as spleen are currently being tested in the clinical setting.

However, there is further need to understand the mechanism of action of such programmed immune interventions. In particular, cell proliferation tumor-homing and tumor-specific cytotoxicity are pathways that are important for therapeutic effects. Thus, the understanding of interaction between the different lines of the immune system and the temporal relationship of the processes is important for further optimizing such therapies. Here, in vivo monitoring and, preferably, imaging is inevitably important [3]. Imaging of the immune system may assist—for example—at optimization of the extent of necessary immune adjuvants, the timing of re-stimulation, and the prediction of the overall therapeutic duration. Nevertheless, possibility of non-invasive imaging of cellular functions might be limited by either the effect size or number of target cells that compose the imaging signal above unspecific background. Moreover, the exact impact of metabolic pathways or surface antigen markers on the success of therapeutic efficacy is not yet clear in many cases.

Since the immune system shows many links to glucose metabolism, imaging of this glucose uptake is hypothesized as an attractive approach for imaging immune processes. For example, mechanisms utilized by T cells upon activation resemble pathways known in malignant cells [4]. Also, B cells show a characteristic increase of glucose metabolism after activation via TLR [5]. Due to the complexity of the interaction between many different cells, imaging of single immune pathways is challenging. Additionally, successful immune interventions are not restricted to one pathway or cell type but rely on a complex network of interaction. This is based on the fact that for all successful immune interventions the strategy of adjuvant stimulation was proved to be fundamentally important [6]. Interestingly, the global effect of immune activation can be imaged by routinely and widely used functional imaging by means of [^18^F]fluoro-2-deoxy-2-d-glucose positron emission tomography (FDG-PET). Although this is widely recognized, typically for discrimination between inflammatory reaction and viable tumor tissue, FDG-PET does not play a significant role for imaging immune processes. One reason might be the different lines of the immune system like the innate and the adaptive immune responses which both are linked to immune effects and changes in glucose metabolism. Thus, the relevance of imaging of splenic FDG uptake at this time is not completely understood. However, implementation of FDG uptake as a possible new marker has several advantages. Glucose metabolism of the immune system might integrate several important pathways reflecting not only activation of T cells but also the immunological network with interaction of the innate and adaptive immune response. Additionally, an advantage of FDG imaging will be the combination of tumor staging and imaging of the immune system within one study. For cancer treatment, T cell responses are the main effector pathways where in turn activation and proliferation might add to the extent of FDG uptake applicable to PET imaging. Many unique advantages of this method can be utilized in imaging of the immune system and might play an important role in clinical and preclinical development of new immunological therapies. Combination of PET with morphological imaging increases the accuracy to align changes in metabolism to the respective organs. By accurate delineation of spleen or lymph node distinction of radioactivity uptake from non-target organs like muscular or gastrointestinal tissue further increases the value of FDG-PET. Therefore, preclinical imaging with FDG-PET/MRI is likely to serve as a powerful method for a variety of new questions in the field of immunotherapy against cancer [7]. In particular, exact in vivo quantification and the possibility of direct translation into clinical practice are highly promising advantages of FDG imaging.

In this study, preclinical FDG-PET was evaluated as an imaging tool to monitor immunological responses upon systemic administration of RNA-based vaccines. In particular, timing and the extent of immune effects were quantified and characterized by ex vivo FACS analysis and correlated to in vivo FDG uptake within the spleen as a target compartment. We could demonstrate the translational potential of FDG-PET as an imaging method for further applications such as development and evaluation of new immunotherapies.

## Methods

### Mice

Female BALB/c mice (6–8 weeks) were purchased from Janvier Laboratories (France) and housed under specific pathogen-free conditions in the animal care facility according to the guidelines of the regional animal care committee. All experiments were performed after ethical committee approval in accordance with federal guidelines. Group sizes were *n* = 3–5 throughout the experiments.

### RNA constructs and in vitro transcription

RNA was generated via in vitro transcription as described previously [8]. pST1-gp70-MITD DNA plasmid which carries a mimotope of the H-2L^d^-restricted AH1 (423-431) epitope derived from Moloney murine leukemia virus (MuLV) envelope glycoprotein 70 (gp70) was used to generate gp70 RNA. This mimotope (AH1-A5) includes an amino acid substitution at position five (V/A; SPSYAYHQF AH1-A5). Irrelevant RNA (irr RNA) was generated by using the pST1-empty-MITD plasmid which does not encode a protein.

### RNA-lipoplexes

RNA-lipoplexes (~ 300–400 nm) were formed by gentle mixing of positively charged liposomes (comprised of DOTMA:DOPE) with the negatively charged gp70 RNA with in a ratio 1.3:2 as described earlier [2, 9]. RNA-lipoplexes were always prepared freshly on the day of each application.

### Vaccination strategies

Unless otherwise stated, 40 μg RNA-lipoplexes in 200 μl volume of isotonic NaCl were applied intravenously via the retrobulbar venous plexus. Control mice received only isotonic NaCl (control) or in some experiments the irrelevant RNA.

### Analysis of spleen cells (FACS)

Spleens were excised and single-cell suspensions prepared by manual disintegration through a 40-μm nylon mesh. After washing, cells were stained with anti-CD4, CD8 (BD Pharmingen), CD11c (Miltenyi Biotec), CD19, CD49b, for linage specific gating and with anti-CD25, CD69, or CD86 (BD Pharmingen), to quantify activation status. CD8^+^ T cells recognizing gp70 AH1-A1 were detected with H-2Ld/AH1423-431 (SPSYVYHQF) tetramer kindly provided by the NIH tetramer core facility (Emory University Vaccine Center).

### In vivo imaging

All in vivo imaging experiments were done under isoflurane anesthesia. BALB/c mice were intravenously vaccinated with RNA lipoplexes and imaged on subsequent days by FDG-PET/MRI (Mediso, Budapest, Hungary). After fasting for 2 h, mice were injected intravenously with FDG (7–8 MBq, AAA, Bonn, Germany) followed by a 45-min uptake period. During the last 10 min of the uptake period, mice were anesthetized with 2% isoflurane and MRI measurements (Material Map for coregistration of the PET scan; 3D Gradient Echo External Averaging (GRE-EXT), multi-field of view (FOV); slice thickness: 0.6 mm; TE: 2 ms; TR: 15 ms; flip angle: 25°) were performed followed by a 15-min static PET scan. Experimental conditions, such as schedule, handling, and heating were always kept constant, to reduce variations in blood glucose levels. PET data were reconstructed with Teratomo 3D (4 iterations, 6 subsets, voxel size 0.4 mm), coregistered to the MR.

### Imaging analysis

Image analysis of PET data was done using the PMOD software (version 3.6). Volumes of interest (VOI) were manually drawn on spleen of corresponding MR data. PET data were corrected for radioactive decay and in vivo FDG uptake was calculated as normalized radioactivity = measured radioactivity/injected radioactivity in units of %ID/cc. In order to exclude relevant partial volume effects, five groups of animals with different spleen activation statuses (animals measured 1–4 days after second vaccination and respective controls) were analyzed with partial volume correction (geometric transfer method) [10]. Therefore, a background VOI was defined in addition to the spleen VOI. Using the geometric transfer method, it is assumed that the tracer uptake is homogenously distributed in each VOI. Spill-over effects in the spleen VOI are estimated by convolving the spleen VOI mask by the scanner point spread function (specified in terms of full width at half maximum: 1.5 mm). Thus, the weighted sum of voxels that are overlapping from the background VOI are estimated and divided by the number of voxels within the background VOI. This yields a geometric transformation matrix that is applied to the measured PET data to assess the true regional tracer uptake.

### Ex vivo biodistribution

After euthanization of the animals at indicated time points, the relevant organs were sampled and weighted and radioactivity was measured by an automated gamma counter (Wizard2 2470, PerkinElmer) to calculate the percentage of accumulated activity as injected dose per gram tissue (% ID/g).

### Clinical imaging

FDG PET/CT was offered to patients enrolled in a phase I clinical trial investigating safety of RNA-lipoplexes in advanced melanoma patients for baseline, interim, and end of treatment staging (Lipo-MERIT, NCT02410733). Two patients with baseline scan and interim staging were evaluated in view of changes of spleen FDG uptake in relation to vaccination. PET/CT was performed after a 4–6-h fasting period, resulting in blood glucose level < 130 mg/dl, and application of approximately 2 MBq/kg FDG after a 60–70 min distribution time. Acquisition was conducted by an EARL-certified Philips Gemini-TOF PET/CT with 2–2.5 min per bed position according to clinical routine. Mean SUV was measured in a 3-cm sphere centered within the spleen.

### Statistical analysis

Data were analyzed using Graph Pad Prism software (version 7.0). Data are represented as mean ± standard error of the mean (SEM). Mean values were compared using two-tailed unpaired Student’s *t* test or one-way ANOVA. The level of significance was defined as *p* < 0.05.

## Results

### Evident FDG uptake in spleen upon RNA-based vaccination

**Fig. 1:**
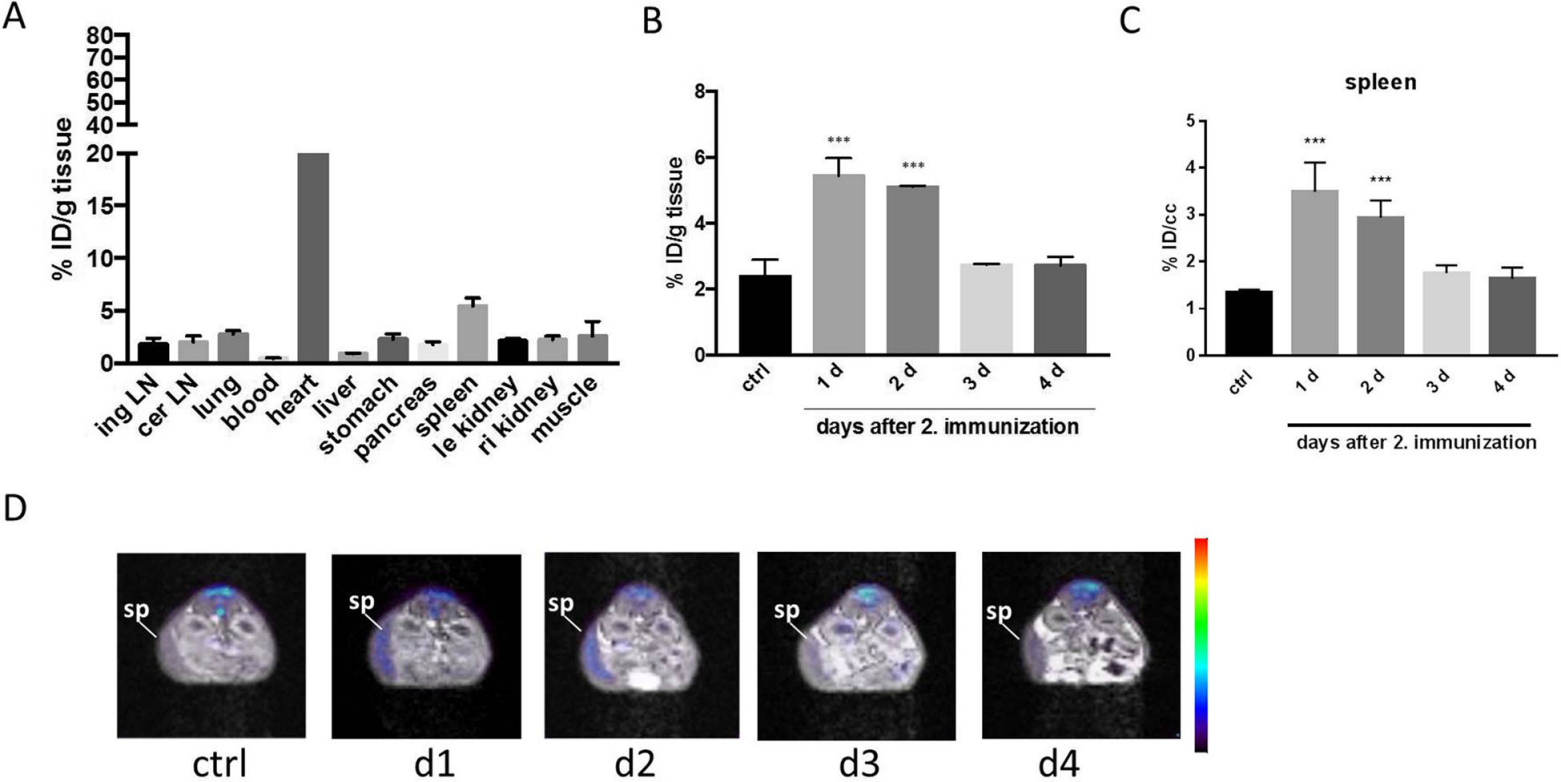
Visualization and quantification of FDG uptake. **a** Ex vivo biodistribution 24 h after second RNA-lipoplex vaccination shows elevated FDG uptake predominantly within the spleen which is the organ with the second highest FDG uptake after myocardium. **b**, **c** Time course of elevated spleen metabolism after the second of two weekly RNA-lipoplex vaccinations FDG uptake returns to normal levels as early as after 72 h. Controls were injected with 0.9% saline. FDG uptake was quantified either by ex vivo biodistribution (**b**) or by in vivo PET imaging (**c**). **d** Visualization of time course of FDG uptake by axial PET/MR slices

In order to track changes upon RNA vaccination, we first evaluated the organ specificity of the FDG uptake. For this purpose, mice were immunized with RNA-lipoplexes encoding the murine leukemia virus (MuLV)-derived protein gp70 in two cycles (day 0 and day 7) and the FDG uptake was measured ex vivo and in vivo by scintillation counting and by PET 24 h after the last vaccination, respectively. Ex vivo biodistribution study demonstrates the response of the immune system after two cycles of RNA vaccination. FDG-uptake within the spleen—after myocardial uptake—reached the second highest uptake from all organs measured (Fig. 1a). Apart from altered uptake in spleen, no changes in other immunologic organs could be detected. In particular, no lymph nodes could be visually detected at the PET scans. Some non-immunologic tissues like muscle or intestine show typically heterogeneous FDG metabolism. Liver as a reference organ showed no differences when compared to control (Additional file 1: Figure S1A). Taking into account the limited resolution of PET and the close anatomic relationship of the mouse spleen to the gastric wall, differentiation might be difficult in small animal imaging without further anatomic information. Therefore, hybrid imaging by means of PET/MRT is a valuable improvement over PET only (Additional file 1: Figure S1B). Of note, the metabolic response to single RNA vaccination on day 1 was also measurable by an increase in FDG uptake in the spleen though it was not statistically significant (Additional file 2: Figure S2).

In order to gain insight on the temporal course of the FDG uptake, we also measured FDG uptake at different time points (1–4 days) after the second vaccination. Comparison of FDG uptake measurement in vivo by PET and by ex vivo means of scintillation counting showed the high accuracy of the method (Fig. 1b, c). Interestingly, the time kinetics revealed a rapid increase of splenic FDG uptake 1 day (24 h) after the second vaccination, which was also evident on day 2 (48 h). The uptake however returned to baseline on day 3 and remained at the level of baseline till the last days of measurement (day 4). Implementation of partial volume correction on FDG uptake within the spleen showed slightly higher uptake (approximately 25%) but exactly the same temporal course (Additional file 3: Figure S3).

#### T cell responses and FDG uptake upon RNA-based vaccination

**Fig. 2:**
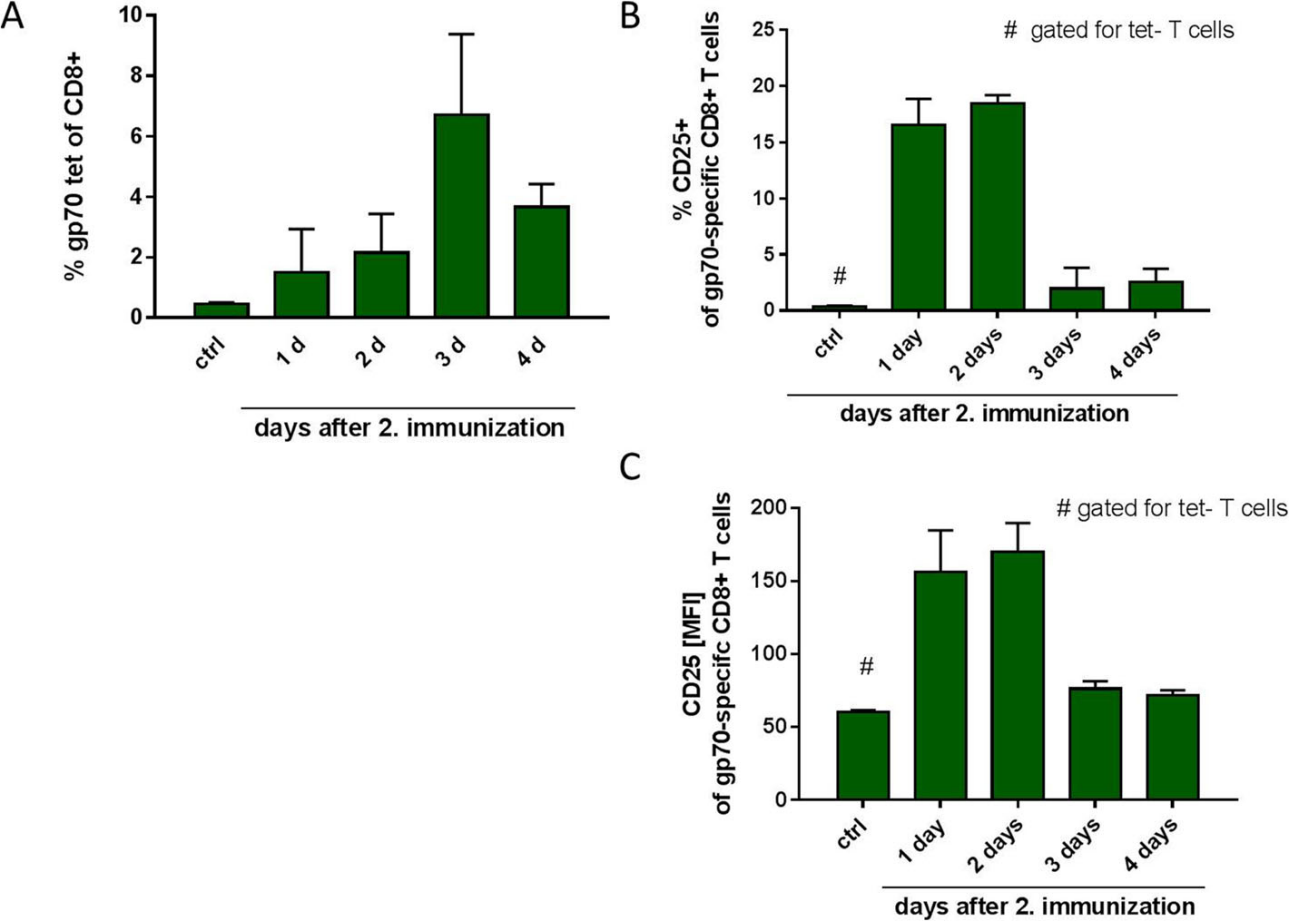
Proliferation and activation of T cells after two cycles of RNA-lipoplex vaccination. **a** Fraction of gp70 tetramer-positive CD8^+^ spleen T cells showing peak proliferation at day three. **b**/**c** Corresponding to the FDG uptake elevation, the activation marker CD25 (MFI/ %) can be detected before the peak of T cell proliferation and returns almost to baseline at day 3

In parallel to the FDG uptake analysis, we also characterized the magnitude of antigen-specific T cell responses as well as activation status of T cells upon two cycles of RNA-lipoplex vaccination as described above. To discriminate the impact of the immune activation of T cells from the impact of T cell proliferation, the time course of antigen-specific T cell proliferation (via gp70 tetramers) as well as T cell activation (via activation marker CD25) were determined in spleen by flow cytometry and then compared to the time course of FDG uptake. The proliferation of gp70-specific CD8^+^ T cells mainly occurred between days 2 and 3 after the vaccination leading to highest frequencies on day 3 followed by a decrease on day 4 (Fig. 2a). On the other hand, the percentage of activated CD25^+^ gp70-specific CD8^+^ T cells was already increased only 1 day after the last vaccination, stayed at the same levels on day 2, and then returned to baseline on days 3 and 4 (Fig. 2b). The expression level of CD25 on these cells also followed a very similar kinetics (Fig. 2c). T cells with other antigen specificities (tetramer-negative population) in control animals or in vaccinated animals did not show any CD25 upregulation (Additional file 4: Figure S4). Indeed, the time course of splenic FDG uptake resembled very much the time course of CD25 expression on T cells. Both are short processes that occur within 24 h after last immunization and are retained for a maximum of 2 days. In contrast, T cell proliferation shows a delayed time course with peak proliferation at day 3. Within the groups, no correlation between FDG uptake and T cell proliferation was evident (data not shown).

**Fig. 3:**
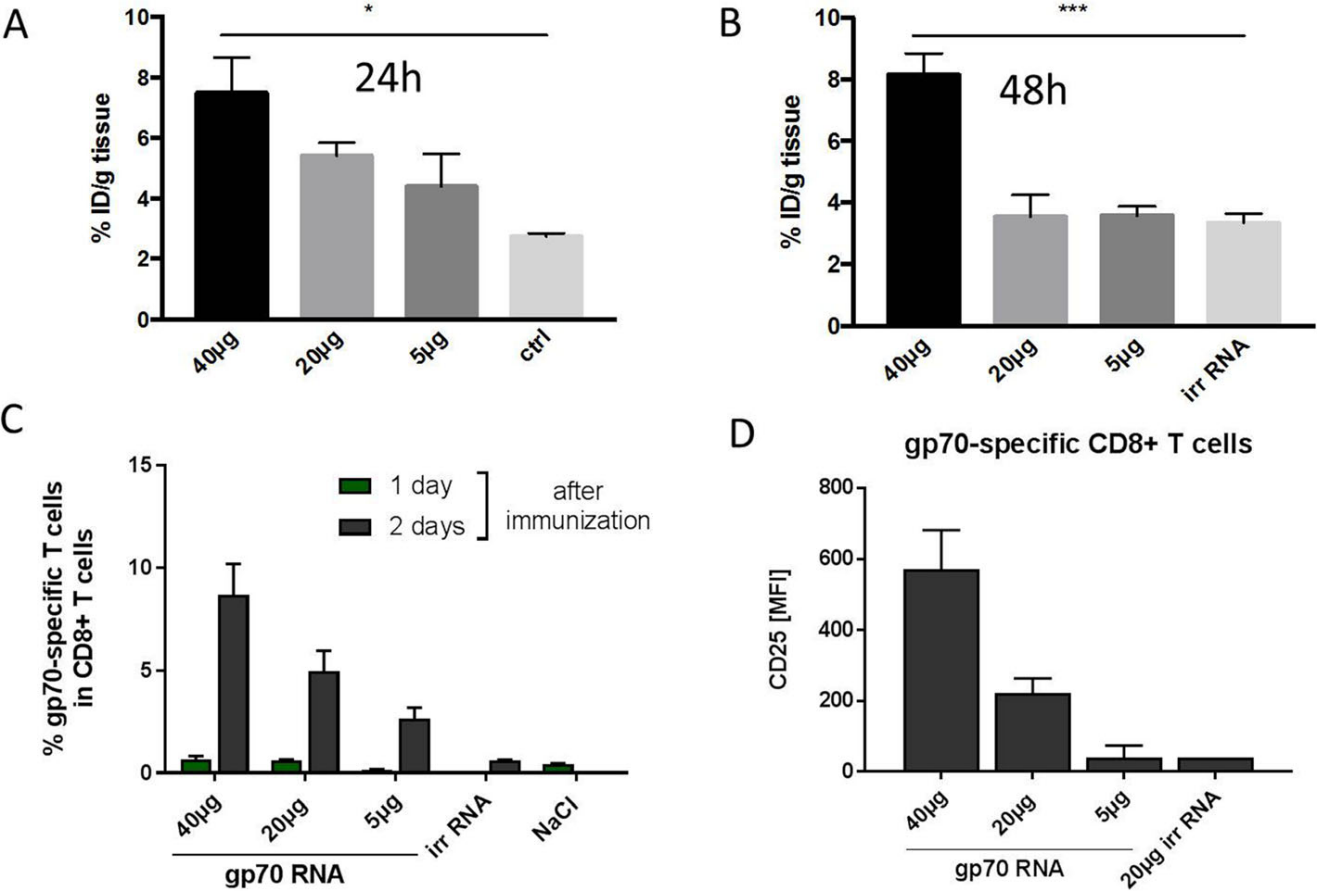
Dose dependency of the immune response after 2 cycle of vaccination as measured by spleen FDG uptake and FACS analysis. Spleen FDG uptake determined by ex vivo biodistribution 24 h (**a**) and 48 h (**b**). A clear dose response is more evident after 24 h, indicating the major role of rapid activating processes. **c** Dose response for T cell proliferation at days 1 and 2. **d** Dose response of CD25 expression on gp70-specific CD8^+^ cells (day 2)

We next studied the effect of RNA dose on the FDG uptake by immunizing mice with various dose of RNA-lipoplexes (40, 20, and 5 μg) in two cycles as described above and investigated the FDG uptake as well as the frequency and activation status of antigen-specific CD8^+^ T cells. FDG uptake clearly followed a dose response when measured 24 h after the last vaccination (Fig. 3a); however, only 40 μg RNA-lipoplex group showed significant FDG uptake at 48 h (Fig. 3b). In terms of antigen-specific T cell proliferation, higher RNA-lipoplex amounts led to higher percentages of antigen-specific CD8^+^ T cells providing also strong evidence for a dose-response relationship (Fig. 3c). Although the frequencies were higher 2 days after the last vaccination compared to those 1 day after, the dose response could be clearly observed. CD25 expression on antigen-specific CD8^+^ T cells also followed a similar trend with higher RNA doses leading to higher CD25 expression (Fig. 3d).

### The effect of non-antigen specific early activation of the immune system on FDG uptake

**Fig. 4:**
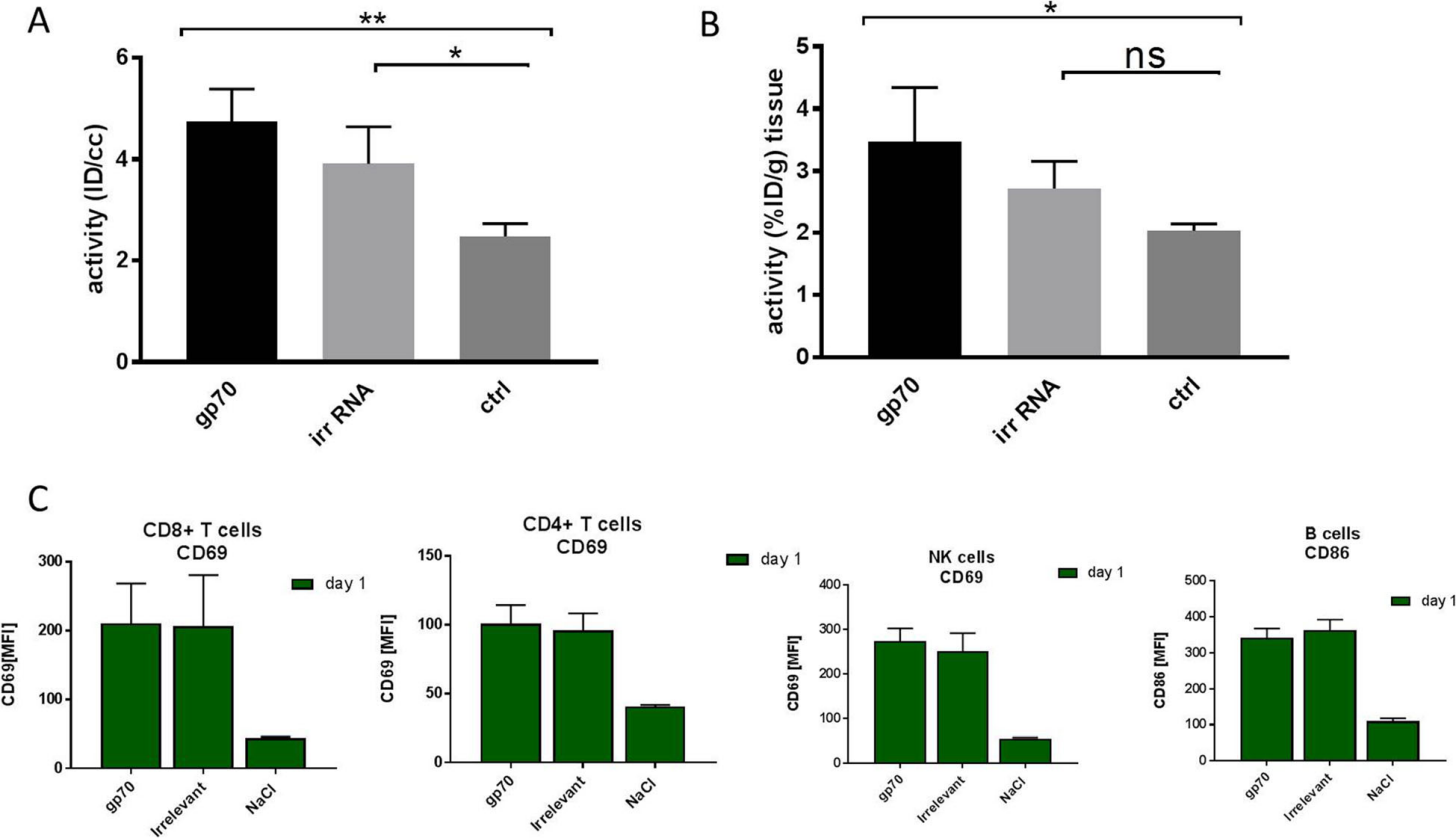
Comparison of FDG uptake in spleen 24 h after two vaccination cycles of gp70 encoding RNA-lipoplexes and irrelevant RNA-lipoplexes not encoding protein information (**a** in vivo PET; **b** ex vivo biodistribution). **c** Increased levels of activation markers CD69 and CD86 were observed in lymphocyte populations and NK cells with RNA irrespective of content 24 h after the second vaccination cycle

To take into account the relationship between non-antigen specific activation of the immune environment in spleen and the FDG uptake, we took the advantage of non-invasive imaging again and applied it 24 h after two cycles of RNA-lipoplex vaccination. As control, we included irrelevant RNA (irr RNA) which exhibits the same adjuvanting structural properties but does not encode for a protein. Thus, the same TLR pathways will be activated with both RNA-lipoplexes but proliferation of gp70 T cells cannot occur after irr RNA vaccination. FDG uptake in spleen was strongly elevated in both the gp70 RNA and the irrelevant RNA groups, although antigen-specific vaccination with gp70 RNA led to slightly higher FDG uptake compared to irr RNA (Fig. 4a, b). Again, close similarity was seen when FDG uptake is quantified from in vivo PET (Fig. 4a) and when quantified by ex vivo gamma counting (Fig. 4b). Statistical analysis reached significance over control for both RNA vaccinations only at the in vivo quantification in line with slightly higher effects after gp70 RNA vaccination. The additional time points for days 2 and 3 are below statistical significance in both groups (Additional file 5: Figure S5).

The self-adjuvanting activity of RNA was evident independent of the antigen as reveled by strong upregulation of activation marker CD69 on T and NK cells as well as CD86 on B cells 24 h after vaccination with either gp70 or irr RNA (Fig. 4c). The expression of these activation markers did not differ between gp70 and irr RNA and returned to steady-state levels 48 h after vaccination (data not shown). Of note, the upregulation of such activation markers was also dose dependent (Additional file 6: Figure S6) and was evident even after single vaccination (Additional file 7: Figure S7). These results point out that the activated immune environment in the spleen upon RNA vaccination mainly contributes to the observed increase in FDG uptake. In contrast, macrophages did not show changes in the activation marker CD86 (data not shown).

### Clinical FDG PET/CT imaging

**Fig. 5:**
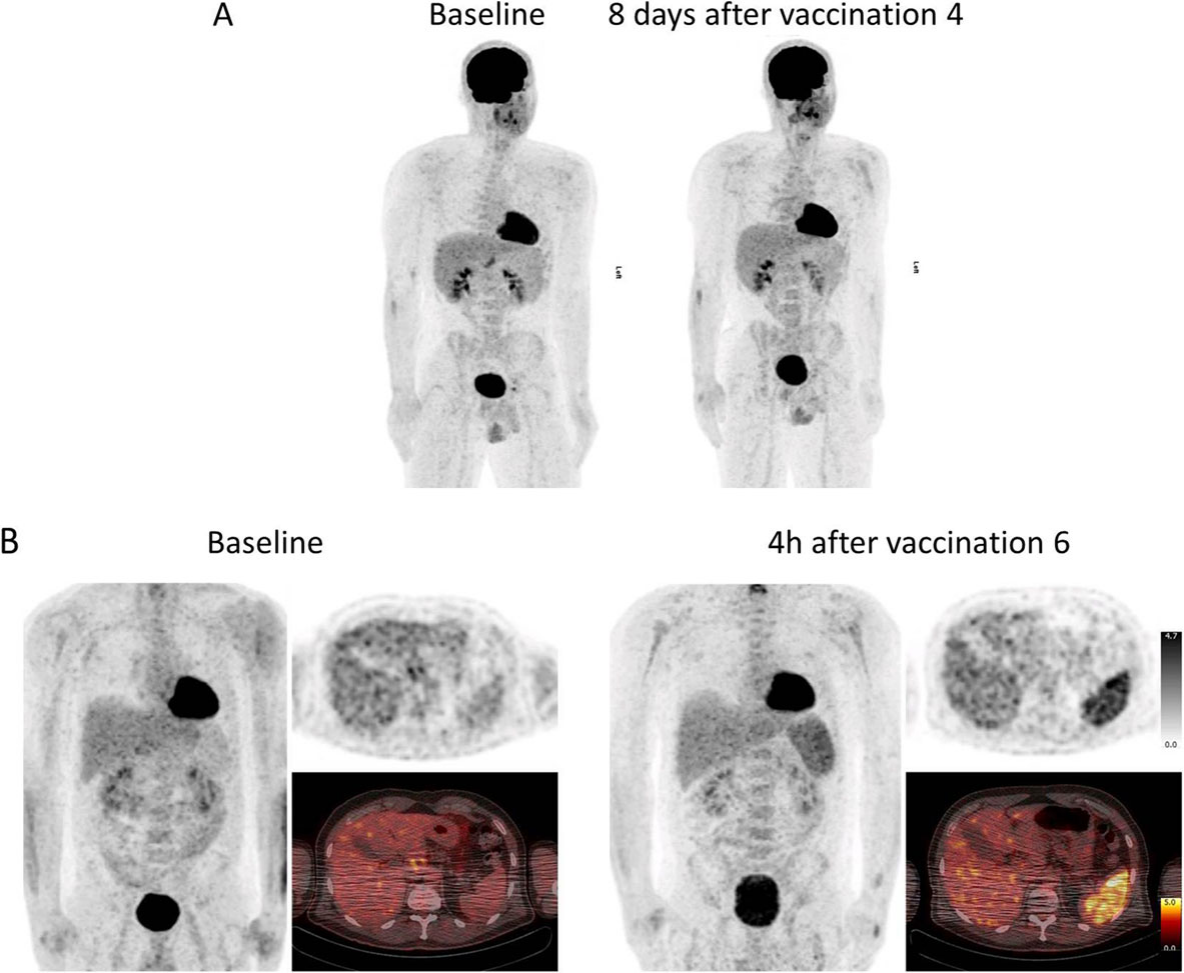
Two clinical cases scanned before and during participation in a clinical vaccination study (LipoMerit). **a** One patient did not show alteration in FDG metabolism during the study when imaging was performed 8 days after the last vaccination cycle. **b** One other patient showed more than two times increased FDG uptake within the spleen when imaging was acquired 4 h after a vaccination cycle

One patient with interim staging 8 days after the fourth vaccination showed normal FDG distribution throughout the body. Spleen uptake was lower in relation to liver both for baseline and interim scans. Quantification showed typical SUV values, unchanged during the course of vaccination. In contrast, one patient with interim staging on the day of the sixth vaccination (time of FDG application at 3 h after vaccination) showed more than twofold increased, homogeneous spleen uptake in comparison to baseline scan (SUV mean at baseline: 1,7; SUV mean at interim staging: 3,6; Fig. 5).

## Discussion

The principle potential of the immune system for targeting malignant cells has been known for many decades. Moreover, recent advances in pharmacological interventions have led to an emerging field of new therapeutic approaches. Among them, targeted RNA delivery is exceptionally promising. On the one hand, RNA displays properties leading to general activation of the immune system, resembling pathways of adjuvants necessary for vaccines for infectious diseases. On the other hand, when targeted delivery is achieved, encoded information is translated and presented to T cells, which results in proliferation of cytotoxic T cells directed towards the intended target. For example, systemic application of slightly negatively charged RNA-lipoplexes (lipoplex) was shown to not only maximize spleen targeting but also achieve strong RNA uptake is antigen-presenting cells such as dendritic cells. T cells primed with those antigen-presenting dendritic cells are then able to migrate and accumulate to tumor sites and attack the tumor by recognizing their specific antigen [2].

Nevertheless, induction of meaningful CD8^+^ T cell proliferation is challenging. One important basis seems the interaction between innate immune system and the adaptive immune response. For example, interferon alpha—commonly expressed by APCs upon TLR stimulation in the context of RNA virus infection [11]—has been shown to act as a highly relevant adjuvant for peptide vaccination against gp100 [12]. A remarkable synergistic effect of adjuvant immune stimulation by a combination of several approaches combining cholera toxin (subunit B), aluminum hydroxide gel, and CpG oligo deoxynucleotide on induction of Muc1-directed T cells was shown in a B16 melanoma mouse model [13]. A large number of pattern-recognition receptors are known, where activation of the innate immune system modulates adaptive responses [14]. Moreover, in the setting of RNA-lipoplexes, TLR7 and Type I interferon (IFNα) pathways activated by RNA also play a role in the complex interaction between innate and adaptive immune system resulting in antigen non-specific activation of various immune cells in the spleen [2]. Thus, non-specific activation of the immune system is also likely to play a key role in determination of the (later) effect of the adaptive immune system against cancer.

By preclinical investigation of the effects of RNA-lipoplex vaccination on glucose metabolism, we could demonstrate that imaging of immune response by FDG-PET/MRI is possible. In particular, we allocate changes in FDG uptake in immune cells mainly towards the rapid activating processes. Further increase of FDG uptake by proliferating T cells is likely, but size of this additional effect remained small without reaching clear statistical significance. In particular, FDG uptake did not show differences over contral at later time points when peak of T cell proliferation was detected by FACS analysis. Lipoplexes serve as carrier of RNA, that otherwise would not reach their immunologic target after intravenous application due to rapid degradation. Therefore, RNA alone would not be able to serve as activator of the immune system. As expected from the inability to activate the immune system also, lipoplexes alone do not influence the FDG spleen uptake (Additional file 8: Figure S8). Taken together, PET imaging of the spleen revealed several key findings. First, along with the spleen-specific distribution pattern of RNA-lipoplexes, FDG uptake was also localized mainly in the spleen. Second, the FDG uptake preceded the proliferation of antigen-specific T cells, the former occurring within the first 2 days while the latter taking place peaking 3 days after RNA-lipoplex vaccination. Interestingly, the kinetics of activation marker CD25 on antigen-specific CD8^+^ T cells correlated well with that of the FGD uptake. Last but not least, a similar dose dependency both on immune response (proliferation and activation) and on FDG uptake was evident.

An important link between the innate and the adaptive immune system consists of cytokine-induced maturation of dendritic cells which in turn initiate the adaptive immune response [15, 16]. Although not completely understood, it can be hypothesized that T cell proliferation based on a generally activated immunological environment is more prominent than selective stimulation of proliferation pathways [17, 18]. One major limitation of imaging as biomarker is the lack of cellular resolution in comparison to FACS analysis. Therefore, exact knowledge about the different cell types and their respective changes in metabolism is difficult to obtain and the results have to be correlated to ex vivo FACS measurements.

In clinical routine, the most readily available imaging method depicting immune effects is FDG-PET/CT. In cancer tissue, glucose metabolism is typically highly increased, due to metabolic shift to aerobic glycolysis. Similar effects are known for immune cells. For example, in patients suffering from granulomatous inflammation (sarcoidosis), FDG uptake within the spleen was elevated. This correlated with serum interleukin-2 receptor elevation as systemic biomarker of inflammation. Here, systemic immune activation seems to be measureable by imaging of FDG uptake in a single organ [19]. By FDG-PET/CT, a retained immune activation over 1 month after vaccination for human papillomaviruses can be demonstrated in lymph nodes [20]. Imaging of immune effects by FDG might be restricted to extra-tumoral immune cells since FDG metabolism within tumor tissue is dominated by tumor cells and negative correlation between most intra-tumoral immune cells with FDG uptake has been shown [21]. Activating the immune response by anti-CTLA4 or PD1 blockade for melanoma treatment also induces changes in metabolic uptake in immune organs like lymph nodes or spleen [22]. Since the adaptive immune response is mainly linked to these organs, FDG imaging might develop to a specific prognostic biomarker for evaluation of immune activation and treatment response in cancer patients. In individual clinical cases, we show that translation into clinical application is possible and demonstrate remarkable similarities between mice and humans in regard to vaccination-induced changes in glucose metabolism. Also in clinical imaging, it is demonstrated that early immune activation is located dominantly within the spleen. Thus, differences in extent and temporal sequence between immune activation and immune proliferation might be detectable in humans by FDG-PET. In particular, FDG-PET/CT might play a future role for optimization of immunological adjuvants in new immunological therapies and also for individualization of their application in clinical routine.

## Conclusions

FDG-PET imaging is able to visualize early immune activation, which in turn seems a promoting environment for targeted immune processes induced by immune-modulating interventions. Translation to clinical applications is highly promising, since FDG imaging then bears the potential in imaging both the vaccination process and its effect on tumor growth. Therefore, FDG PET bears the potential to sequentially image and quantify immune processes resulting in new applications in individualization of immune therapies by an already widely existing imaging method.

## Additional files

Additional file 1: **Figure S1.** A: Liver as a reference organ showed no differences when compared to control. B: hybrid imaging by means of PET/MRT is a valuable improvement over PET only. St: stomach, s: spleen, i: intestine. (JPG 140 kb)

Additional file 2: **Figure S2.** Initial response of splenic FDG uptake and T cells after a single vaccination (24 h) demonstrating rapid immunological response to RNA-lipoplex, though not statistically significant. (JPG 99 kb)

Additional file 3: **Figure S3.** A: Comparison of in vivo FDG uptake in the spleen with and without partial volume correction together with ex vivo uptake. B: Representative display of MRI (left) and PET (right). Spleen was manually delineated on the MRI and projected towards PET. (JPG 231 kb)

Additional file 4: **Figure S4.** Non-antigen-specific cells (tetramer neg) did not upregulate CD25 expression. (JPG 129 kb)

Additional file 5: **Figure S5.** Comparison of ex vivo FDG spleen uptake after two vaccination cycles with either gp70 encoding and with irrelevant RNA compared to control. (JPG 103 kb)

Additional file 6: **Figure S6.** CD69 activation was also dose dependent. (JPG 155 kb)

Additional file 7: **Figure S7.** Expression of activation marker CD6 on T cells after only one RNA-lipoplex vaccination cycle. (JPG 94 kb)

Additional file 8: **Figure S8.** Balb/c mice were immunized two times (d0 and d7) with 20 μg gp70-LPX, liposomes alone, gp70-RNA or with NaCl (ctrl). FDG was applied i.v. 24 h later, and the accumulation in the spleen, liver, and lung was measured 1 h p.i. *n* = 3 mice/group. (JPG 147 kb)

## References

[1] Sahin U, Derhovanessian E, Miller M, Kloke BP, Simon P, Lower M (2017). Personalized RNA mutanome vaccines mobilize poly-specific therapeutic immunity against cancer. Nature.

[2] Kranz LM, Diken M, Haas H, Kreiter S, Loquai C, Reuter KC (2016). Systemic RNA delivery to dendritic cells exploits antiviral defence for cancer immunotherapy. Nature.

[3] Ehlerding EB, England CG, McNeel DG, Cai W (2016). Molecular imaging of immunotherapy targets in cancer. J Nucl Med.

[4] Chang CH, Curtis JD, Maggi LB, Faubert B, Villarino AV, O'Sullivan D (2013). Posttranscriptional control of T cell effector function by aerobic glycolysis. Cell.

[5] Pektor S, Bausbacher N, Otto G, Lawaczeck L, Grabbe S, Schreckenberger M (2016). Toll like receptor mediated immune stimulation can be visualized in vivo by [18F]FDG-PET. Nucl Med Biol.

[6] van der Burg SH, Arens R, Ossendorp F, van Hall T, Melief CJ (2016). Vaccines for established cancer: overcoming the challenges posed by immune evasion. Nat Rev Cancer.

[7] Diken M, Pektor S, Miederer M (2016). Harnessing the potential of noninvasive in vivo preclinical imaging of the immune system: challenges and prospects. Nanomedicine (London).

[8] Kreiter S, Konrad T, Sester M, Huber C, Tureci O, Sahin U (2007). Simultaneous ex vivo quantification of antigen-specific CD4+ and CD8+ T cell responses using in vitro transcribed RNA. Cancer Immunol Immunother.

[9] Barichello JM, Ishida T, Kiwada H (2010). Complexation of siRNA and pDNA with cationic liposomes: the important aspects in lipoplex preparation. Methods Mol Biol.

[10] Rousset OG, Ma Y, Evans AC (1998). Correction for partial volume effects in PET: principle and validation. J Nucl Med.

[11] Diebold SS, Kaisho T, Hemmi H, Akira S, Reis e Sousa C (2004). Innate antiviral responses by means of TLR7-mediated recognition of single-stranded RNA. Science.

[12] Sikora AG, Jaffarzad N, Hailemichael Y, Gelbard A, Stonier SW, Schluns KS (2009). IFN-alpha enhances peptide vaccine-induced CD8+ T cell numbers, effector function, and antitumor activity. J Immunol.

[13] Lu W, Qiu L, Yan Z, Lin Z, Cao M, Hu C (2015). Cytotoxic T cell responses are enhanced by antigen design involving the presentation of MUC1 peptide on cholera toxin B subunit. Oncotarget.

[14] Iwasaki A, Medzhitov R (2015). Control of adaptive immunity by the innate immune system. Nat Immunol.

[15] Zhang H, Gregorio JD, Iwahori T, Zhang X, Choi O, Tolentino LL (2017). A distinct subset of plasmacytoid dendritic cells induces activation and differentiation of B and T lymphocytes. Proc Natl Acad Sci U S A.

[16] Kadowaki N, Antonenko S, Lau JY, Liu YJ (2000). Natural interferon alpha/beta-producing cells link innate and adaptive immunity. J Exp Med.

[17] Mujal AM, Gilden JK, Gerard A, Kinoshita M, Krummel MF (2016). A septin requirement differentiates autonomous and contact-facilitated T cell proliferation. Nat Immunol.

[18] Ramanathan S, Gagnon J, Ilangumaran S (2008). Antigen-nonspecific activation of CD8+ T lymphocytes by cytokines: relevance to immunity, autoimmunity, and cancer. Arch Immunol Ther Exp.

[19] Kalkanis A, Kalkanis D, Drougas D, Vavougios GD, Datseris I, Judson MA (2016). Correlation of spleen metabolism assessed by 18F-FDG PET with serum interleukin-2 receptor levels and other biomarkers in patients with untreated sarcoidosis. Nucl Med Commun.

[20] Coates EE, Costner PJ, Nason MC, Herrin DM, Conant S, Herscovitch P (2017). Lymph node activation by PET/CT following vaccination with licensed vaccines for human papillomaviruses. Clin Nucl Med.

[21] Na KJ, Choi H. Tumor metabolic features identified by FDG PET correlates with gene networks of immune cell microenvironment in head and neck cancer. J Nucl Med. 2017; 10.2967/jnumed.117.194217. 10.2967/jnumed.117.194217 28588149

[22] Tsai KK, Pampaloni MH, Hope C, Algazi AP, Ljung BM, Pincus L (2016). Increased FDG avidity in lymphoid tissue associated with response to combined immune checkpoint blockade. J Immunother Cancer.

