# Supplementary figures and images for: In vivo imaging of the immune response upon systemic RNA cancer vaccination by FDG-PET
Source: EJNMMI Res. 2018 Aug 15;8:80. doi: 10.1186/s13550-018-0435-z (PMC6093825; doi:10.1186/s13550-018-0435-z)

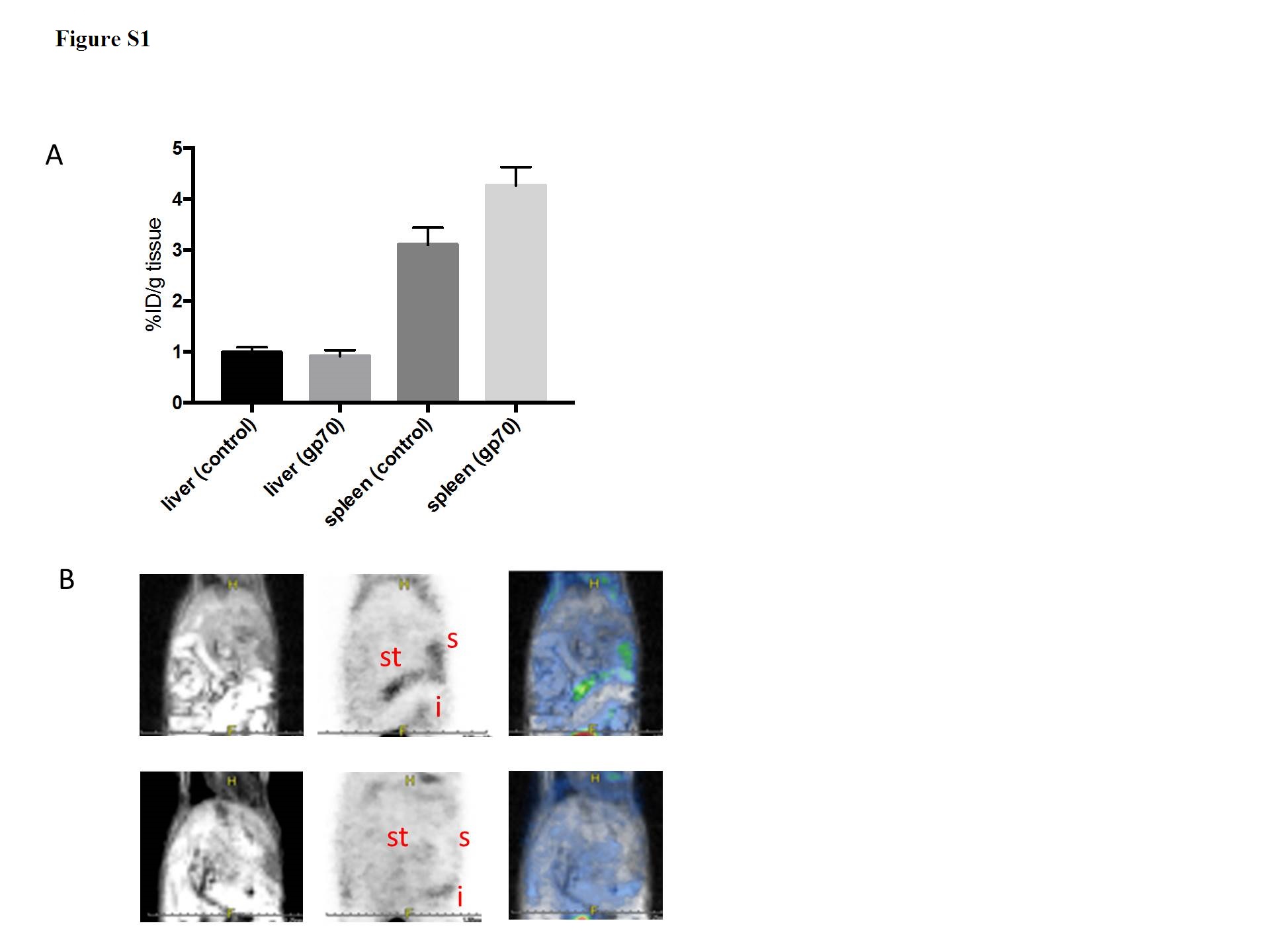

Supplement: Supplementary file 1 — Figure S1. A: Liver as a reference organ showed no differences when compared to control. B: hybrid imaging by means of PET/MRT is a valuable improvement over PET only. St: stomach, s: spleen, i: intestine. (JPG 140 kb) [file 13550_2018_435_MOESM1_ESM.jpg]

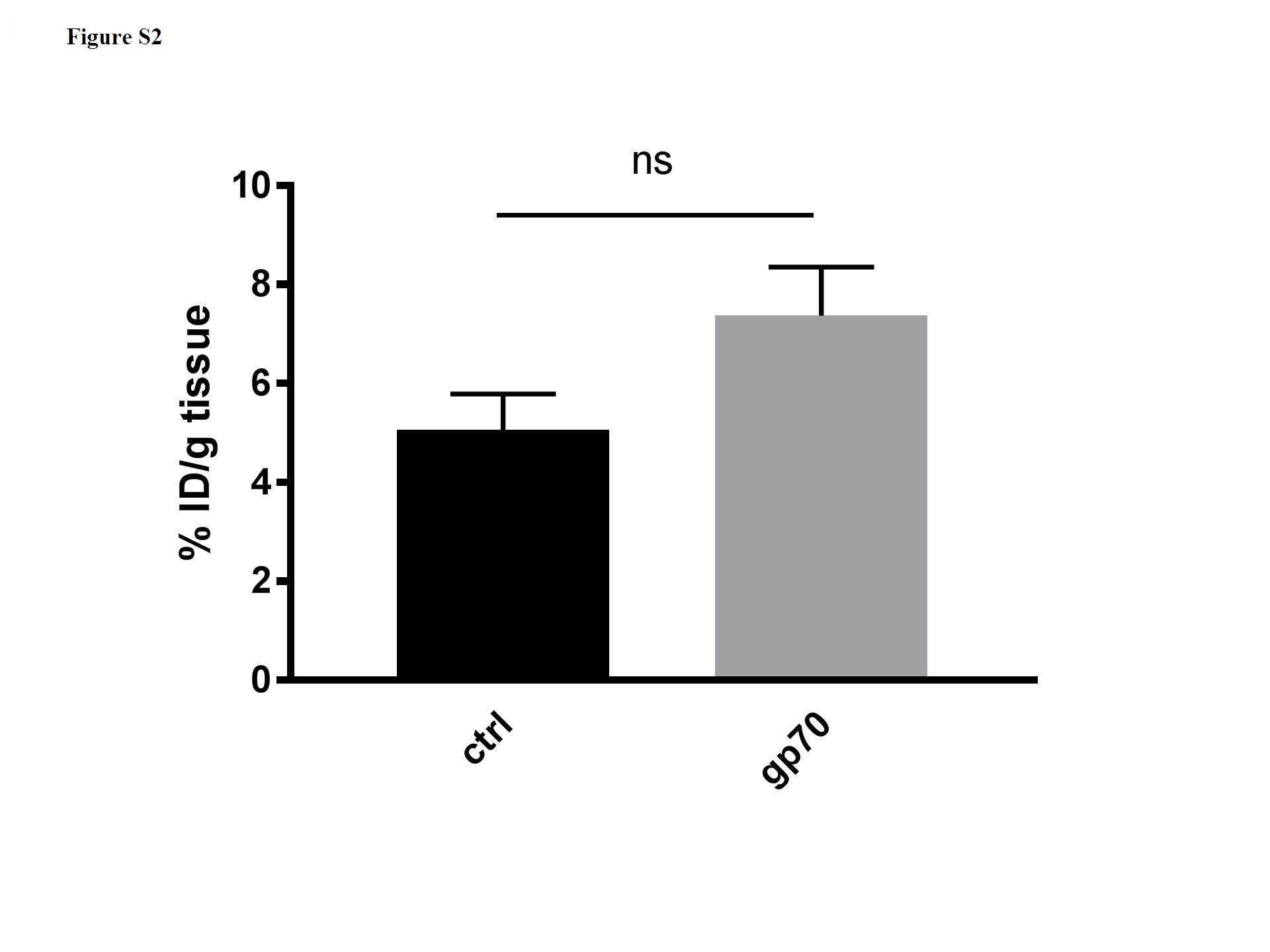

Supplement: Supplementary file 2 — Figure S2. Initial response of splenic FDG uptake and T cells after a single vaccination (24 h) demonstrating rapid immunological response to RNA-lipoplex, though not statistically significant. (JPG 99 kb) [file 13550_2018_435_MOESM2_ESM.jpg]

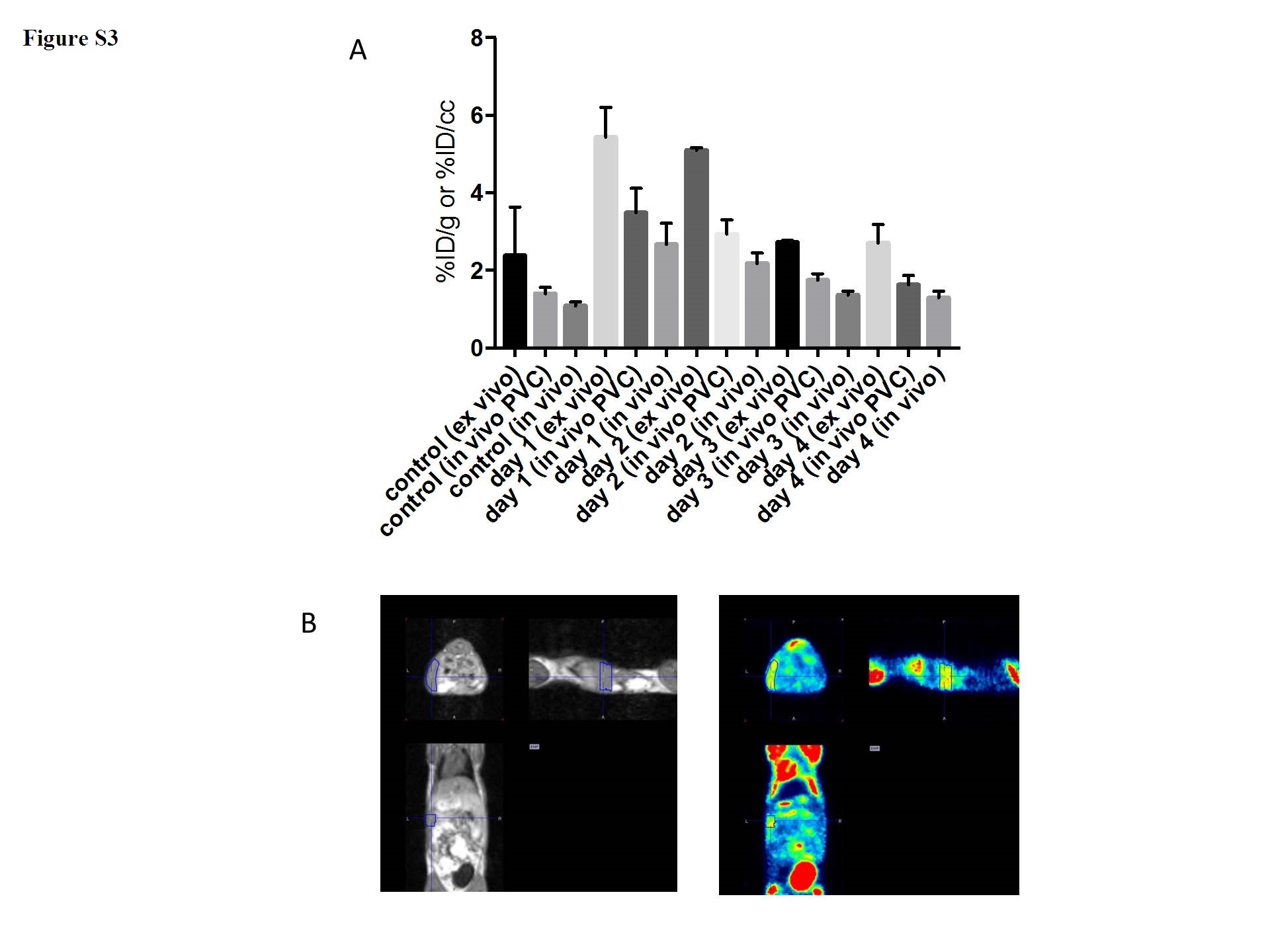

Supplement: Supplementary file 3 — Figure S3. A: Comparison of in vivo FDG uptake in the spleen with and without partial volume correction together with ex vivo uptake. B: Representative display of MRI (left) and PET (right). Spleen was manually delineated on the MRI and projected towards PET. (JPG 231 kb) [file 13550_2018_435_MOESM3_ESM.jpg]

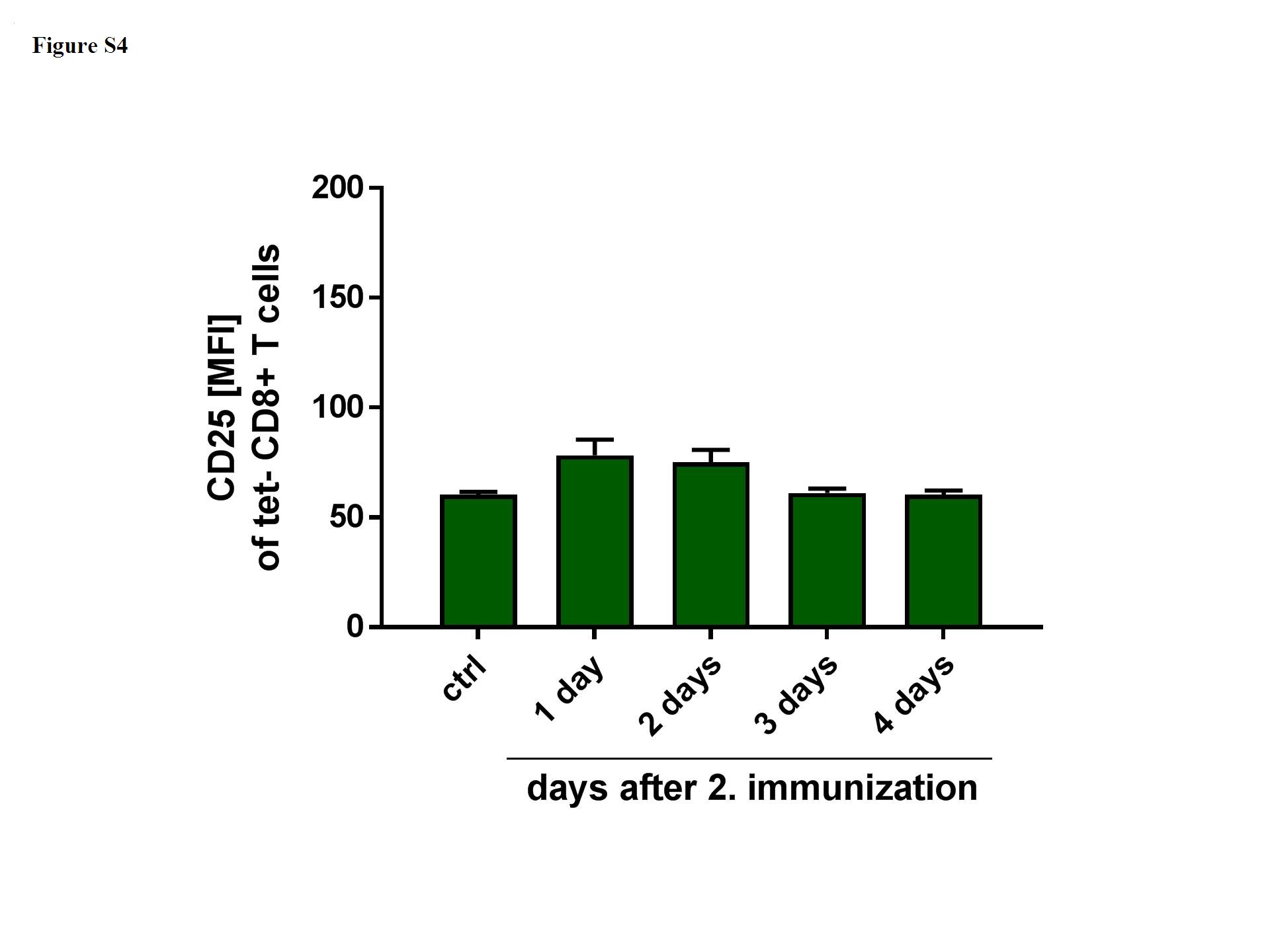

Supplement: Supplementary file 4 — Figure S4. Non-antigen-specific cells (tetramer neg) did not upregulate CD25 expression. (JPG 129 kb) [file 13550_2018_435_MOESM4_ESM.jpg]

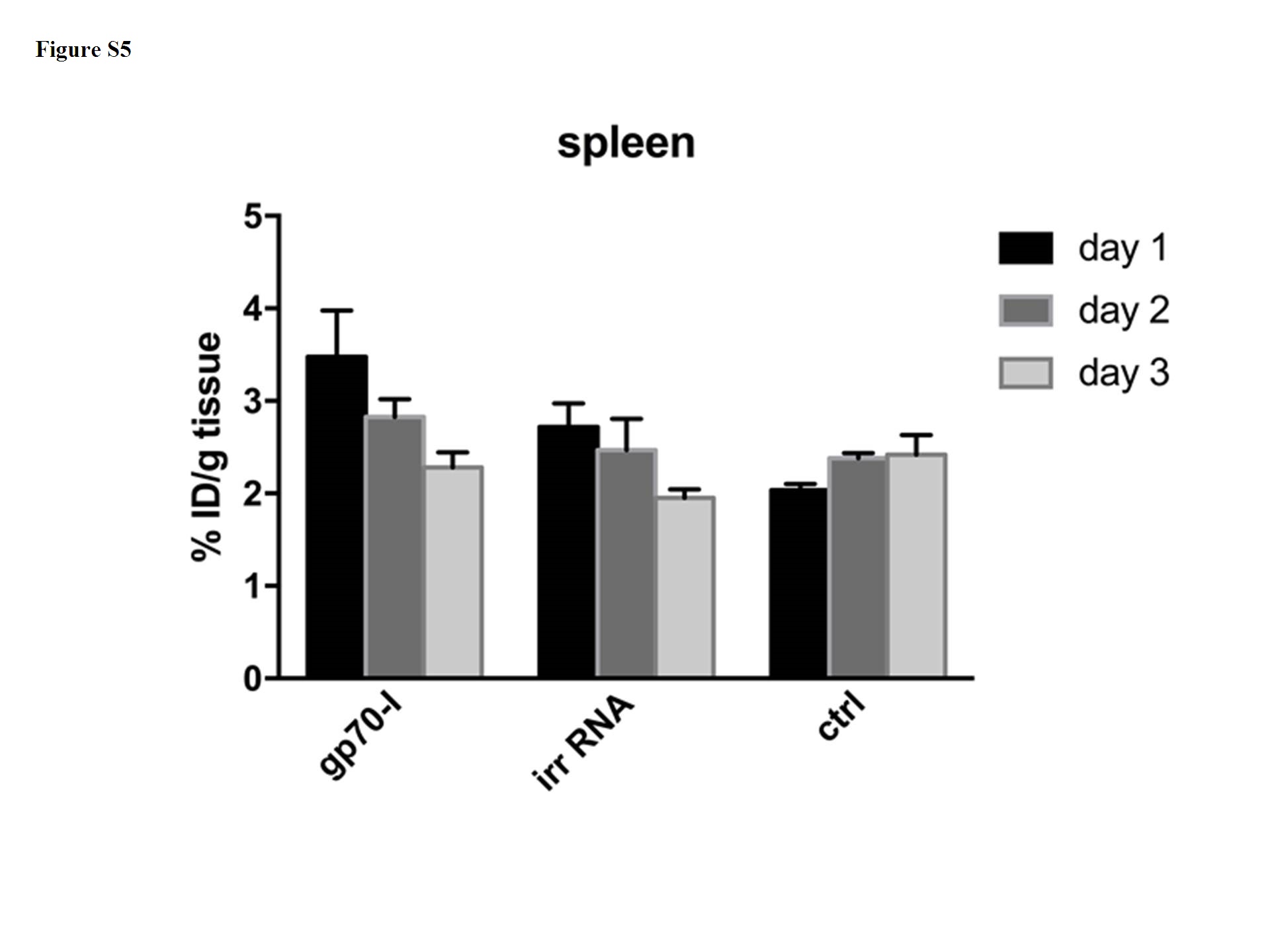

Supplement: Supplementary file 5 — Figure S5. Comparison of ex vivo FDG spleen uptake after two vaccination cycles with either gp70 encoding and with irrelevant RNA compared to control. (JPG 103 kb) [file 13550_2018_435_MOESM5_ESM.jpg]

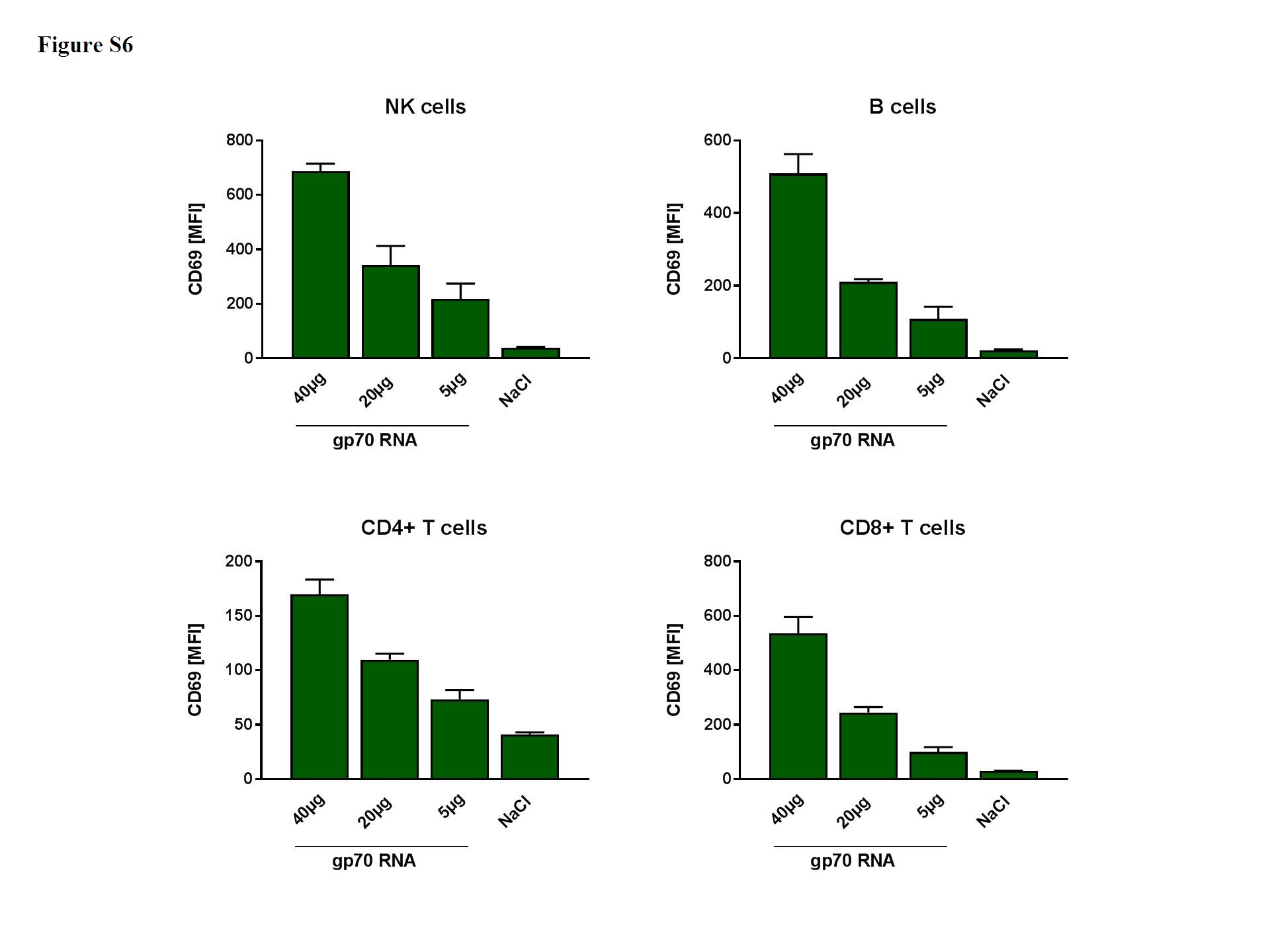

Supplement: Supplementary file 6 — Figure S6. CD69 activation was also dose dependent. (JPG 155 kb) [file 13550_2018_435_MOESM6_ESM.jpg]

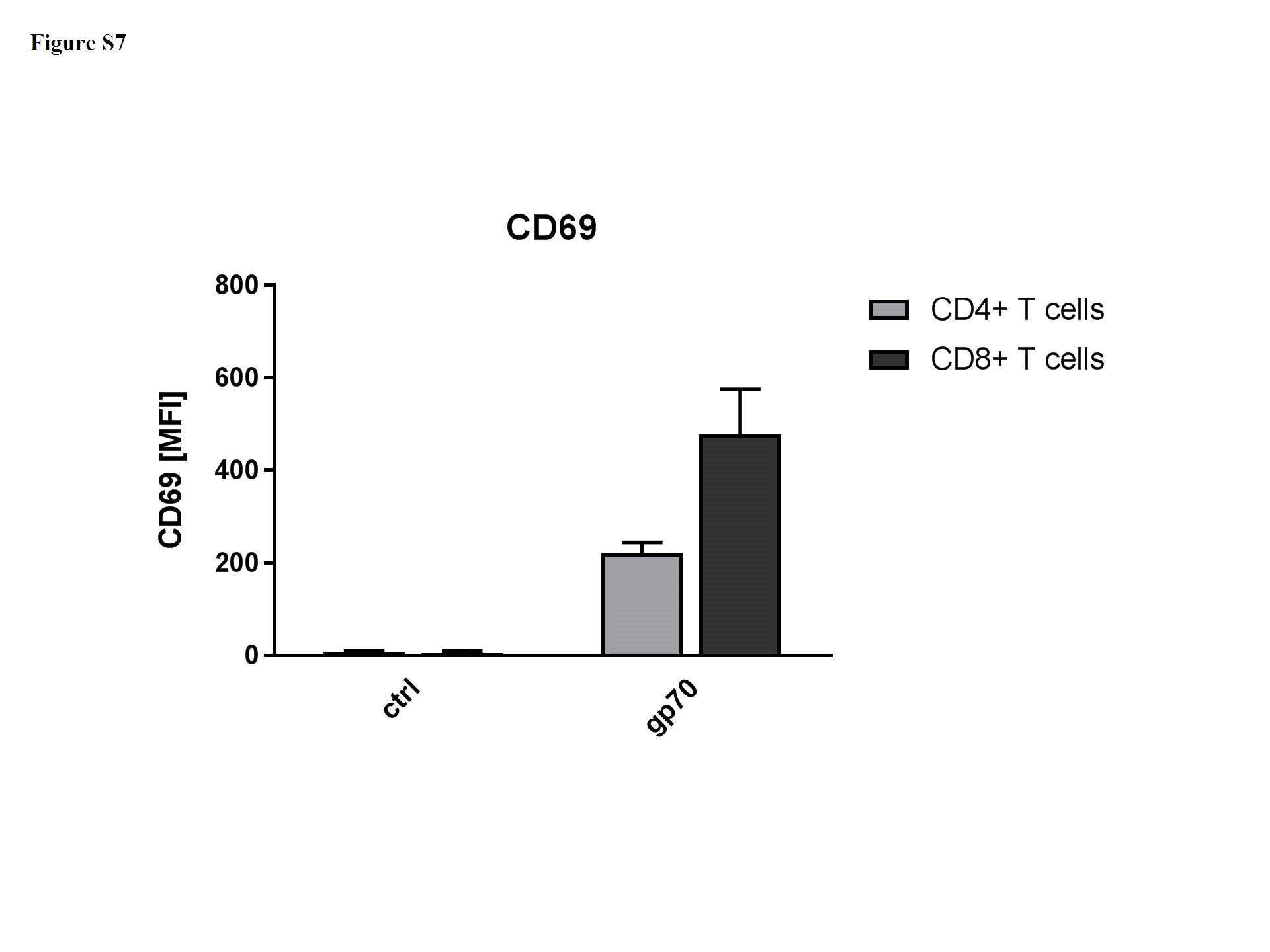

Supplement: Supplementary file 7 — Figure S7. Expression of activation marker CD6 on T cells after only one RNA-lipoplex vaccination cycle. (JPG 94 kb) [file 13550_2018_435_MOESM7_ESM.jpg]

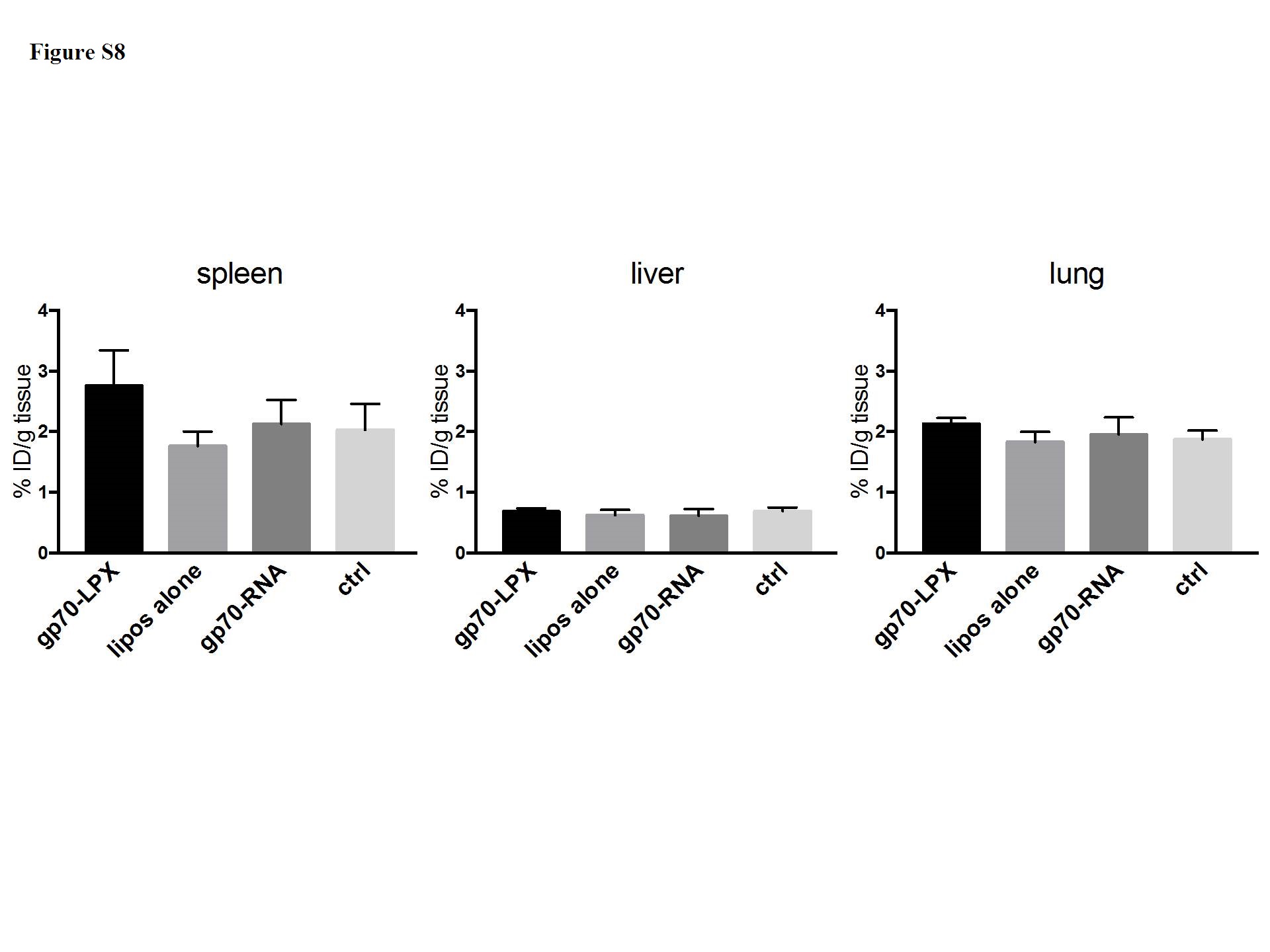

Supplement: Supplementary file 8 — Figure S8. Balb/c mice were immunized two times (d0 and d7) with 20 μg gp70-LPX, liposomes alone, gp70-RNA or with NaCl (ctrl). FDG was applied i.v. 24 h later, and the accumulation in the spleen, liver, and lung was measured 1 h p.i. n = 3 mice/group. (JPG 147 kb) [file 13550_2018_435_MOESM8_ESM.jpg]
